# 5-Chloro-6-hy­droxy-7,8-dimethyl­chroman-2-one

**DOI:** 10.1107/S1600536811029345

**Published:** 2011-07-30

**Authors:** Scott A. Cameron, Shailesh K. Goswami, Lyall R. Hanton, C. John McAdam, Stephen C. Moratti, Jim Simpson

**Affiliations:** a Department of Chemistry, University of Otago, PO Box 56, Dunedin, New Zealand

## Abstract

In the title mol­ecule, C_11_H_11_ClO_3_, the fused pyran ring adopts a half-chair conformation. In the crystal, inter­molecular O—H⋯O hydrogen bonds link mol­ecules into chains along [100]. These chains are inter­connected by weak inter­molecular C—H⋯O contacts which generate *R*_2_^2^(8) ring motifs, forming sheets parallel to (001). Tetra­gonal symmetry generates an equivalent motif along *b*. Furthermore, the sheets are linked along the *c* axis by offset π–π stacking inter­actions involving the benzene rings of adjacent mol­ecules [with centroid–centroid distances of 3.839 (2) Å], together with an additional weak C—H⋯O hydrogen bond, resulting in an overall three-dimensional network.

## Related literature

For the synthesis of the starting materials, see: Fieser & Ardao (1956 ▶); Bishop *et al.* (1963 ▶). For related structures, see: Budzianowski & Katrusiak (2002 ▶); Goswami *et al.* (2011 ▶). For standard bond lengths, see Allen *et al.* (1987 ▶). For hydrogen-bond motifs, see: Bernstein *et al.* (1995 ▶).
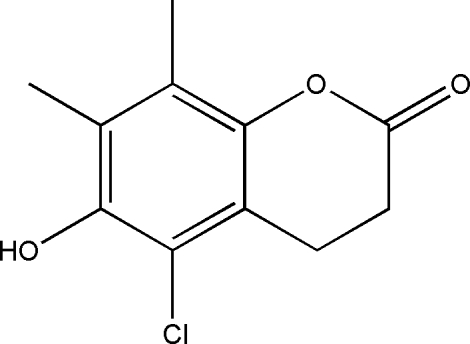


## Experimental

### 

#### Crystal data

C_11_H_11_ClO_3_
*M* *_r_* = 226.65
Tetragonal, 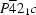

*a* = 16.1375 (6) Å
*c* = 7.5887 (6) Å
*V* = 1976.24 (19) Å^3^
*Z* = 8
Mo *K*α radiation
μ = 0.37 mm^−1^
*T* = 89 K
0.40 × 0.07 × 0.05 mm

#### Data collection

Bruker APEXII CCD area-detector diffractometer
Absorption correction: multi-scan (*SADABS*; Bruker 2009 ▶) *T*_min_ = 0.792, *T*_max_ = 1.00
22360 measured reflections
2030 independent reflections
1618 reflections with *I* > 2σ(*I*)
*R*_int_ = 0.095

#### Refinement

*R*[*F* ^2^ > 2σ(*F* ^2^)] = 0.050
*wR*(*F* ^2^) = 0.143
*S* = 1.07
2030 reflections
141 parameters
H atoms treated by a mixture of independent and constrained refinement
Δρ_max_ = 0.33 e Å^−3^
Δρ_min_ = −0.39 e Å^−3^
Absolute structure: Flack (1983 ▶), 859 Friedel pairs
Flack parameter: −0.04 (12)

### 

Data collection: *APEX2* (Bruker 2009 ▶); cell refinement: *SAINT* (Bruker 2009 ▶); data reduction: *SAINT*; program(s) used to solve structure: *SHELXS97* (Sheldrick, 2008 ▶); program(s) used to refine structure: *SHELXL97* (Sheldrick, 2008 ▶) and *TITAN2000* (Hunter & Simpson, 1999 ▶); molecular graphics: *SHELXTL* (Sheldrick, 2008 ▶) and *Mercury* (Macrae *et al.*, 2008 ▶); software used to prepare material for publication: *SHELXL97*, *enCIFer* (Allen *et al.*, 2004 ▶), *PLATON* (Spek, 2009 ▶) and *publCIF* (Westrip, 2010 ▶).

## Supplementary Material

Crystal structure: contains datablock(s) I, global. DOI: 10.1107/S1600536811029345/lh5289sup1.cif

Structure factors: contains datablock(s) I. DOI: 10.1107/S1600536811029345/lh5289Isup2.hkl

Supplementary material file. DOI: 10.1107/S1600536811029345/lh5289Isup3.cml

Additional supplementary materials:  crystallographic information; 3D view; checkCIF report

## Figures and Tables

**Table 1:** Hydrogen-bond geometry (Å, °)

*D*—H⋯*A*	*D*—H	H⋯*A*	*D*⋯*A*	*D*—H⋯*A*
C8—H8*A*⋯O9^i^	0.99	2.55	3.463 (5)	154
C7—H7*B*⋯O1^ii^	0.99	2.64	3.588 (5)	160
C7—H7*B*⋯O9^ii^	0.99	2.68	3.357 (5)	126
O4—H4*O*⋯O9^iii^	0.78 (4)	2.12 (5)	2.748 (4)	137 (4)
C8—H8*B*⋯O4^iv^	0.99	2.39	3.328 (5)	158

Symmetry codes: (i) 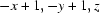; (ii) 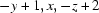; (iii) 
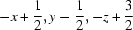
; (iv) 
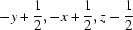
.

**Fig. 1.**
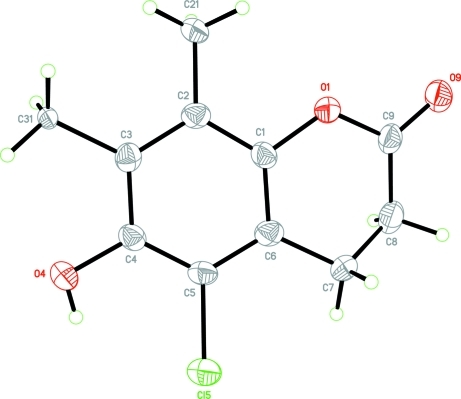
The molecular structure of (I) showing ellipsoids drawn at the 50% probability level.

**Fig. 2.**
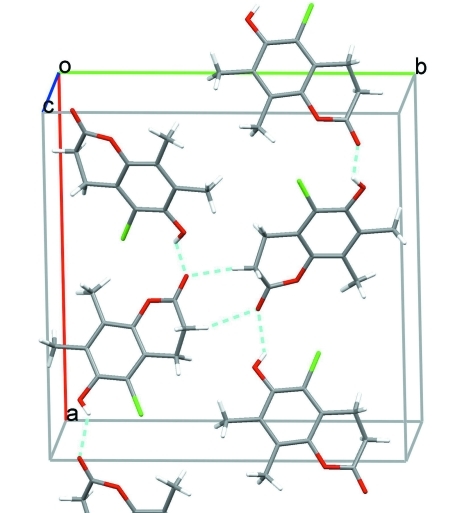
The (0 0 1) layer of (I). Dashed lines show O–H···O hydrogen bonds and C–H···O interactions.

**Fig. 3.**
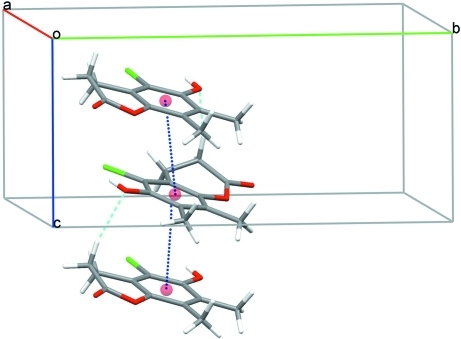
The π..π stacking interactions in the structure of (I) with C–H···O interactions drawn as dashed lines.

**Fig. 4.**
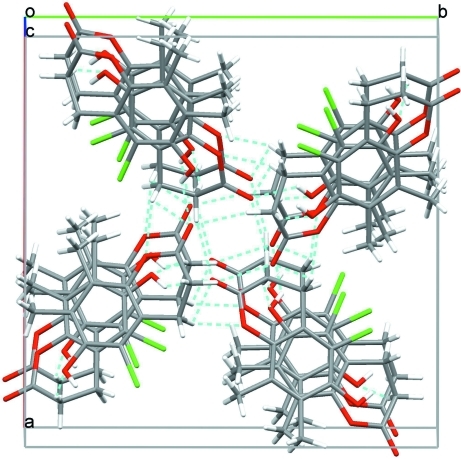
Crystal packing of (I) viewed along the c axis showing the three- dimensional network.

## supplementary crystallographic information

### Comment

The title compound (I) was isolated as an intermediate during the synthesis of redox-active quinone monomers currently of interest to us in our electro-mechanical actuator programme.

Compound (I), Fig 1, consists of a chromanone unit with an OH substituent at C4, a chloro substituent at C5 and methyl substituents on C2 and C3. The fused C1/C6-C9/O1 ring is in a half-chair conformation. Bond distances (Allen *et al.*, 1987) and angles are normal and similar to those in closely related structures (Budzianowski & Katrusiak, 2002; Goswami *et al.*, 2011).

Classical O4—H4O···O9^iii^ hydrogen bonds link molecules into chains along *a*. These chains are interconnected by weak C8—H8A···O9^i^ contacts which generate *R*^2^_2_(8) ring motifs (Bernstein *et al.*, 1995) forming sheets in (0 0 1), Fig 2. Tetragonal symmetry generates an equivalent motif along b. These sheets are stacked along *c* by offset π–π stacking interactions involving the benzene rings of adjacent molecules with centroid to centroid distances of 3.839 (2) Å together with an additional C8–H8B···O4^iv^ hydrogen bond, Fig 3, resulting in a three dimensional network structure, Fig 4.

### Experimental

The title compound was synthesized in three steps. In the first step trimethyl-*p*-hydroquinone (Fieser & Ardao, 1956) (15.2 g, 100 mmol) was oxidized using sodium dichromate (10.8 g, 41 mmol) in acetic acid (50 ml). The product was characterized using NMR spectroscopy and the data were consistent with reported data of trimethyl-*p*-benzoquinone. The second step (chlorination) is an alternative to the existing literature (Bishop *et al.*, 1963). Trimethyl-*p*-benzoquinone (10 g, 67 mmol) was added to conc. hydrochloric acid (100 ml) with vigorous stirring. The resulting suspension was heated to reflux for 3 hr. After dilution with water the solid was filtered out and re-dissolved in aqueous acetic acid. Aqueous sodium dichromate (10 g, 38 mmol) was added in portions. After the mixture had stood for 15 min, a yellow solid was precipitated by dilution with water. Crystallization from ethanol-water solution gave a yellow material, m.p. 337-338K; (lit. m.p. 337-338K). In the final step, a solution of methyl malonate (5.7 g, 43 mmol) in dry MeOH (25 ml) was refluxed for one hour with finely powdered MgOMe (3.85 g, 70 mmol). A solution of chlorotrimethyl-*p*-quinone (4 g, 21 mmol) in dry MeOH (25 ml) was added dropwise to the refluxing solution and reflux continued for 13 hr. The solid was removed from the cooled mixture, washed with ether and carefully mixed with HCl (10%, 50 ml) and stirred at 283K to remove impurities. The yellow solid product (3 g) was filtered out and dissolved in acetone and stirred with dil. hydrochloric acid (100 ml). The resulting white suspension was then refluxed for 5 hr. The solution was cooled and extracted with ether (3 × 30 mL) and the combined organic extracts washed with brine, dried (MgSO_4_) and evaporated. To the crude residue in toluene (60 ml), 4-methylbenzenesulfonic acid (0.47 g, 27 mmol) was added with stirring, and the mixture then refluxed. After 12 hr, the nearly colourless solution was cooled to room temp. and extracted with EtOAc (3 × 30 ml). The organic extract was washed with sat. aqueous NaHCO_3_ and the aqueous layer back-extracted once with EtOAc (30 ml). The combined organic extracts were washed with brine and dried over MgSO_4_. X-ray quality crystals of the title compound, 5-chloro-6-hydroxy-7,8-dimethylchroman-2-one were obtained from EtOAc/hexane (1.76 g, 80%): m.p. 139–41°C; FT—IR cm^-1^ 1777 (O—C=O); ^1^H-NMR (400 MHz, CDCl_3_): δ 2.20 (s, 3H), 2.33 (s, 3H), 2.74 (t, J = 8 Hz, 2H), 3.02 (t, J = 8 Hz, 2H), 5.5 (s, 1H); ^13^C-NMR (100 MHz, CDCl_3_): δ 12.0, 12.6, 22.0, 28.6,115.1, 117.8, 123.8, 125.0, 143.8, 145.9, 165.3.

### Refinement

The OH hydrogen atom was located in a difference Fourier map and refined freely with *U*_iso_ = 1.2*U*_eq_ (O). Methyl and methylene H-atoms were refined using a riding model with d(C—H) = 0.98 Å, *U*_iso_=1.5*U*_eq_ (C) for methyl and 0.99 Å, *U*_iso_=1.2*U*_eq_ (C) for methylene.

### Crystal data

**Table:**

C_11_H_11_ClO_3_	*D*_x_ = 1.524 Mg m^−^^3^
*M**_r_* = 226.65	Mo *K*α radiation, λ = 0.71073 Å
Tetragonal, *P*42_1_*c*	Cell parameters from 1966 reflections
Hall symbol: P -4 2n	θ = 2.5–20.6°
*a* = 16.1375 (6) Å	µ = 0.37 mm^−^^1^
*c* = 7.5887 (6) Å	*T* = 89 K
*V* = 1976.24 (19) Å^3^	Needle, colourless
*Z* = 8	0.40 × 0.07 × 0.05 mm
*F*(000) = 944	

### Data collection

**Table:**

Bruker APEXII CCD area-detector diffractometer	2030 independent reflections
Radiation source: fine-focus sealed tube	1618 reflections with *I* > 2σ(*I*)
graphite	*R*_int_ = 0.095
ω scans	θ_max_ = 26.4°, θ_min_ = 3.9°
Absorption correction: multi-scan (*SADABS*; Bruker 2009)	*h* = −20→20
*T*_min_ = 0.792, *T*_max_ = 1.00	*k* = −20→19
22360 measured reflections	*l* = −9→8

### Refinement

**Table:**

Refinement on *F*^2^	Secondary atom site location: difference Fourier map
Least-squares matrix: full	Hydrogen site location: inferred from neighbouring sites
*R*[*F*^2^ > 2σ(*F*^2^)] = 0.050	H atoms treated by a mixture of independent and constrained refinement
*wR*(*F*^2^) = 0.143	*w* = 1/[σ^2^(*F*_o_^2^) + (0.0781*P*)^2^ + 0.6384*P*] where *P* = (*F*_o_^2^ + 2*F*_c_^2^)/3
*S* = 1.07	(Δ/σ)_max_ < 0.001
2030 reflections	Δρ_max_ = 0.33 e Å^−^^3^
141 parameters	Δρ_min_ = −0.39 e Å^−^^3^
0 restraints	Absolute structure: Flack (1983), 859 Friedel pairs
Primary atom site location: structure-invariant direct methods	Flack parameter: −0.04 (12)

### Special details

**Table:**

Geometry. All e.s.d.'s (except the e.s.d. in the dihedral angle between two l.s. planes) are estimated using the full covariance matrix. The cell e.s.d.'s are taken into account individually in the estimation of e.s.d.'s in distances, angles and torsion angles; correlations between e.s.d.'s in cell parameters are only used when they are defined by crystal symmetry. An approximate (isotropic) treatment of cell e.s.d.'s is used for estimating e.s.d.'s involving l.s. planes.
Refinement. Refinement of *F*^2^ against ALL reflections. The weighted *R*-factor *wR* and goodness of fit *S* are based on *F*^2^, conventional *R*-factors *R* are based on *F*, with *F* set to zero for negative *F*^2^. The threshold expression of *F*^2^ > σ(*F*^2^) is used only for calculating *R*-factors(gt) *etc*. and is not relevant to the choice of reflections for refinement. *R*-factors based on *F*^2^ are statistically about twice as large as those based on *F*, and *R*- factors based on ALL data will be even larger.

### Fractional atomic coordinates and isotropic or equivalent isotropic displacement parameters (Å^2^)

**Table:**

	*x*	*y*	*z*	*U*_iso_*/*U*_eq_	
O1	0.29181 (15)	0.46995 (15)	0.9081 (4)	0.0286 (6)	
C1	0.2457 (2)	0.3970 (2)	0.8839 (5)	0.0246 (8)	
C2	0.1624 (2)	0.4026 (2)	0.9338 (5)	0.0246 (8)	
C21	0.1277 (2)	0.4829 (2)	1.0015 (6)	0.0295 (9)	
H2A	0.1709	0.5132	1.0655	0.044*	
H2B	0.0812	0.4715	1.0810	0.044*	
H2C	0.1083	0.5165	0.9021	0.044*	
C3	0.1126 (2)	0.3318 (2)	0.9168 (5)	0.0243 (8)	
C31	0.02056 (19)	0.3310 (2)	0.9744 (4)	0.0166 (7)	
H3A	−0.0132	0.3587	0.8847	0.025*	
H3B	0.0148	0.3602	1.0870	0.025*	
H3C	0.0019	0.2736	0.9879	0.025*	
C4	0.1472 (2)	0.2595 (2)	0.8461 (5)	0.0239 (8)	
O4	0.09528 (17)	0.19372 (16)	0.8261 (4)	0.0308 (7)	
H4O	0.121 (3)	0.162 (3)	0.769 (6)	0.037*	
C5	0.2308 (2)	0.2574 (2)	0.8003 (5)	0.0256 (8)	
Cl5	0.27140 (6)	0.16405 (6)	0.72670 (15)	0.0370 (3)	
C6	0.2815 (2)	0.3256 (2)	0.8168 (5)	0.0253 (8)	
C7	0.3714 (2)	0.3268 (2)	0.7691 (6)	0.0294 (9)	
H7A	0.3812	0.2882	0.6699	0.035*	
H7B	0.4045	0.3074	0.8709	0.035*	
C8	0.3994 (2)	0.4131 (3)	0.7170 (6)	0.0347 (9)	
H8A	0.4607	0.4149	0.7194	0.042*	
H8B	0.3815	0.4239	0.5943	0.042*	
C9	0.3668 (2)	0.4805 (2)	0.8318 (6)	0.0279 (9)	
O9	0.40023 (17)	0.54650 (16)	0.8556 (4)	0.0348 (7)	

### Atomic displacement parameters (Å^2^)

**Table:**

	*U*^11^	*U*^22^	*U*^33^	*U*^12^	*U*^13^	*U*^23^
O1	0.0223 (13)	0.0216 (13)	0.0419 (16)	0.0005 (11)	0.0013 (11)	−0.0028 (11)
C1	0.0241 (19)	0.0182 (17)	0.032 (2)	−0.0010 (15)	−0.0035 (15)	0.0018 (16)
C2	0.0260 (19)	0.0201 (18)	0.0278 (19)	0.0045 (16)	−0.0020 (16)	0.0018 (15)
C21	0.027 (2)	0.0183 (19)	0.043 (2)	−0.0007 (16)	−0.0001 (18)	−0.0065 (17)
C3	0.0210 (19)	0.0255 (18)	0.0263 (18)	0.0006 (15)	−0.0035 (15)	0.0032 (16)
C31	0.0134 (16)	0.0129 (16)	0.0235 (18)	−0.0005 (13)	−0.0005 (13)	0.0001 (14)
C4	0.0262 (19)	0.0184 (17)	0.0270 (18)	−0.0008 (14)	−0.0024 (15)	0.0024 (15)
O4	0.0281 (15)	0.0227 (14)	0.0418 (17)	−0.0042 (12)	−0.0004 (13)	−0.0031 (12)
C5	0.0287 (19)	0.0177 (17)	0.031 (2)	0.0034 (15)	−0.0003 (17)	0.0001 (15)
Cl5	0.0364 (6)	0.0255 (5)	0.0492 (6)	0.0042 (4)	−0.0028 (5)	−0.0048 (5)
C6	0.0259 (19)	0.0222 (19)	0.0279 (19)	0.0049 (15)	−0.0044 (15)	0.0016 (15)
C7	0.0213 (17)	0.0247 (18)	0.042 (2)	0.0044 (14)	−0.0024 (17)	−0.0012 (18)
C8	0.0212 (19)	0.042 (2)	0.041 (2)	−0.0005 (16)	−0.0001 (17)	−0.002 (2)
C9	0.0193 (18)	0.025 (2)	0.039 (2)	0.0000 (15)	−0.0036 (16)	0.0061 (17)
O9	0.0273 (14)	0.0227 (14)	0.0543 (19)	−0.0014 (12)	−0.0046 (14)	0.0067 (13)

### Geometric parameters (Å, °)

**Table:**

O1—C9	1.353 (4)	C4—O4	1.361 (4)
O1—C1	1.404 (4)	C4—C5	1.394 (5)
C1—C6	1.386 (5)	O4—H4O	0.78 (4)
C1—C2	1.399 (5)	C5—C6	1.377 (5)
C2—C3	1.402 (5)	C5—Cl5	1.735 (3)
C2—C21	1.503 (5)	C6—C7	1.495 (5)
C21—H2A	0.9800	C7—C8	1.517 (5)
C21—H2B	0.9800	C7—H7A	0.9900
C21—H2C	0.9800	C7—H7B	0.9900
C3—C4	1.401 (5)	C8—C9	1.489 (6)
C3—C31	1.549 (5)	C8—H8A	0.9900
C31—H3A	0.9800	C8—H8B	0.9900
C31—H3B	0.9800	C9—O9	1.207 (4)
C31—H3C	0.9800		
			
C9—O1—C1	121.6 (3)	C5—C4—C3	120.1 (3)
C6—C1—C2	123.6 (3)	C4—O4—H4O	104 (3)
C6—C1—O1	121.6 (3)	C6—C5—C4	122.2 (3)
C2—C1—O1	114.8 (3)	C6—C5—Cl5	120.0 (3)
C1—C2—C3	118.2 (3)	C4—C5—Cl5	117.8 (3)
C1—C2—C21	120.4 (3)	C5—C6—C1	116.8 (3)
C3—C2—C21	121.3 (3)	C5—C6—C7	124.3 (3)
C2—C21—H2A	109.5	C1—C6—C7	118.9 (3)
C2—C21—H2B	109.5	C6—C7—C8	111.3 (3)
H2A—C21—H2B	109.5	C6—C7—H7A	109.4
C2—C21—H2C	109.5	C8—C7—H7A	109.4
H2A—C21—H2C	109.5	C6—C7—H7B	109.4
H2B—C21—H2C	109.5	C8—C7—H7B	109.4
C4—C3—C2	119.1 (3)	H7A—C7—H7B	108.0
C4—C3—C31	118.9 (3)	C9—C8—C7	114.4 (3)
C2—C3—C31	122.1 (3)	C9—C8—H8A	108.7
C3—C31—H3A	109.5	C7—C8—H8A	108.7
C3—C31—H3B	109.5	C9—C8—H8B	108.7
H3A—C31—H3B	109.5	C7—C8—H8B	108.7
C3—C31—H3C	109.5	H8A—C8—H8B	107.6
H3A—C31—H3C	109.5	O9—C9—O1	116.5 (4)
H3B—C31—H3C	109.5	O9—C9—C8	125.1 (4)
O4—C4—C5	123.3 (3)	O1—C9—C8	118.3 (3)
O4—C4—C3	116.6 (3)		
			
C9—O1—C1—C6	15.2 (5)	C3—C4—C5—Cl5	176.0 (3)
C9—O1—C1—C2	−165.2 (3)	C4—C5—C6—C1	0.8 (6)
C6—C1—C2—C3	0.7 (6)	Cl5—C5—C6—C1	−177.1 (3)
O1—C1—C2—C3	−178.9 (3)	C4—C5—C6—C7	−179.8 (4)
C6—C1—C2—C21	−178.5 (4)	Cl5—C5—C6—C7	2.2 (5)
O1—C1—C2—C21	1.9 (5)	C2—C1—C6—C5	−0.2 (6)
C1—C2—C3—C4	−1.8 (5)	O1—C1—C6—C5	179.3 (3)
C21—C2—C3—C4	177.4 (3)	C2—C1—C6—C7	−179.6 (4)
C1—C2—C3—C31	177.9 (3)	O1—C1—C6—C7	0.0 (6)
C21—C2—C3—C31	−2.9 (6)	C5—C6—C7—C8	152.5 (4)
C2—C3—C4—O4	−177.5 (3)	C1—C6—C7—C8	−28.2 (5)
C31—C3—C4—O4	2.8 (5)	C6—C7—C8—C9	42.6 (5)
C2—C3—C4—C5	2.5 (6)	C1—O1—C9—O9	177.5 (4)
C31—C3—C4—C5	−177.2 (3)	C1—O1—C9—C8	1.3 (5)
O4—C4—C5—C6	177.9 (4)	C7—C8—C9—O9	153.5 (4)
C3—C4—C5—C6	−2.0 (6)	C7—C8—C9—O1	−30.7 (5)
O4—C4—C5—Cl5	−4.0 (5)		

### Hydrogen-bond geometry (Å, °)

**Table:**

*D*—H···*A*	*D*—H	H···*A*	*D*···*A*	*D*—H···*A*
C8—H8A···O9^i^	0.99	2.55	3.463 (5)	154.
C7—H7B···O1^ii^	0.99	2.64	3.588 (5)	160.
C7—H7B···O9^ii^	0.99	2.68	3.357 (5)	126.
O4—H4O···O9^iii^	0.78 (4)	2.12 (5)	2.748 (4)	137 (4)
C8—H8B···O4^iv^	0.99	2.39	3.328 (5)	158.

Symmetry codes: (i) −*x*+1, −*y*+1, *z*; (ii) −*y*+1, *x*, −*z*+2; (iii) −*x*+1/2, *y*−1/2, −*z*+3/2; (iv) −*y*+1/2, −*x*+1/2, *z*−1/2.

## References

[bb1] Allen, F. H., Johnson, O., Shields, G. P., Smith, B. R. & Towler, M. (2004). *J. Appl. Cryst.* **37**, 335–338.

[bb2] Allen, F. H., Kennard, O., Watson, D. G., Brammer, L., Orpen, A. G. & Taylor, R. (1987). *J. Chem. Soc. Perkin Trans. 2*, pp. S1–19.

[bb3] Bernstein, J., Davis, R. E., Shimoni, L. & Chang, N.-L. (1995). *Angew. Chem. Int. Ed. Engl.* **34**, 1555–1573.

[bb4] Bishop, C. A., Porter, R. F. & Tong, L. K. J. (1963). *J. Am. Chem. Soc.* **85**, 3991–3998.

[bb5] Bruker (2009). *APEX2*, *SAINT* and *SADABS* Bruker AXS Inc., Madison, Wisconsin, USA.

[bb6] Budzianowski, A. & Katrusiak, A. (2002). *Acta Cryst.* B**58**, 125–133. 10.1107/s0108768101017955 11818660

[bb7] Fieser, L. F. & Ardao, M. I. (1956). *J. Am. Chem. Soc.* **78**, 774–781.

[bb8] Flack, H. D. (1983). *Acta Cryst.* A**39**, 876–881.

[bb9] Goswami, S. K., Hanton, L. R., McAdam, C. J., Moratti, S. C. & Simpson, J. (2011). *Acta Cryst.* E**67**, o1566–o1567. 10.1107/S1600536811019982 PMC3151777 21836979

[bb10] Hunter, K. A. & Simpson, J. (1999). *TITAN2000* University of Otago, New Zealand.

[bb11] Macrae, C. F., Bruno, I. J., Chisholm, J. A., Edgington, P. R., McCabe, P., Pidcock, E., Rodriguez-Monge, L., Taylor, R., van de Streek, J. & Wood, P. A. (2008). *J. Appl. Cryst.* **41**, 466–470.

[bb12] Sheldrick, G. M. (2008). *Acta Cryst.* A**64**, 112–122. 10.1107/S0108767307043930 18156677

[bb13] Spek, A. L. (2009). *Acta Cryst.* D**65**, 148–155. 10.1107/S090744490804362X PMC2631630 19171970

[bb14] Westrip, S. P. (2010). *J. Appl. Cryst.* **43**, 920–925.

